# Modified micro-pulling-down method for preparation of phosphor–glass composite scintillators

**DOI:** 10.1039/d6ra05762d

**Published:** 2026-10-30

**Authors:** Herbert Kindl, František Zajíc, Eduard Leitner, Karol Bartosiewicz, Vítězslav Jarý, Monika Kotyková, Romana Kučerková, Alena Beitlerová, Paweł Socha, Anton Markovskyi, Ryszard Buczyński, Martin Nikl

**Affiliations:** a Institute of Physics of the Czech Academy of Sciences Cukrovarnická 10 Prague 16200 Czech Republic kindlh@fzu.cz zajicf@fzu.cz; b University of Chemistry and Technology Prague Technická 5 Prague 16628 Czech Republic; c Faculty of Nuclear Sciences and Physical Engineering, Czech Technical University in Prague Břehová 7 Prague 11519 Czech Republic; d Lukasiewicz Research Network, Institute of Microelectronics and Photonics Al. Lotników 32/46 02-668 Warsaw Poland; e Faculty of Physics, University of Warsaw Ul. Pasteura 5 02-093 Warsaw Poland

## Abstract

Single-crystal scintillators offer superior performance, but their high production cost stimulates the development of alternative cost-effective materials for ionizing radiation detection. A composite material consisting of scintillating particles embedded in a glass matrix represents a promising low-cost alternative, while providing mechanical stability, optical transparency, and shapeability. We present an optimized hot-zone design for the micro-pulling-down method enabling rapid preparation of glass composite rods with embedded Y_3_Al_5_O_12_:Ce (YAG:Ce) micro particles, with a rod geometry that is reproducible across independently grown runs. The scintillation and photoluminescence measurements of the prepared samples reveal higher radioluminescence intensity under identical excitation conditions compared to samples prepared by the melt-quenching method. This approach enables systematic studies focused on the development of high-performance and low-cost composite scintillators.

## Abstract 2

We present an optimized hot-zone design for the micro-pulling-down method enabling rapid preparation of glass composite rods with embedded Y_3_Al_5_O_12_:Ce (YAG:Ce) micro particles.

## Graphical abstract

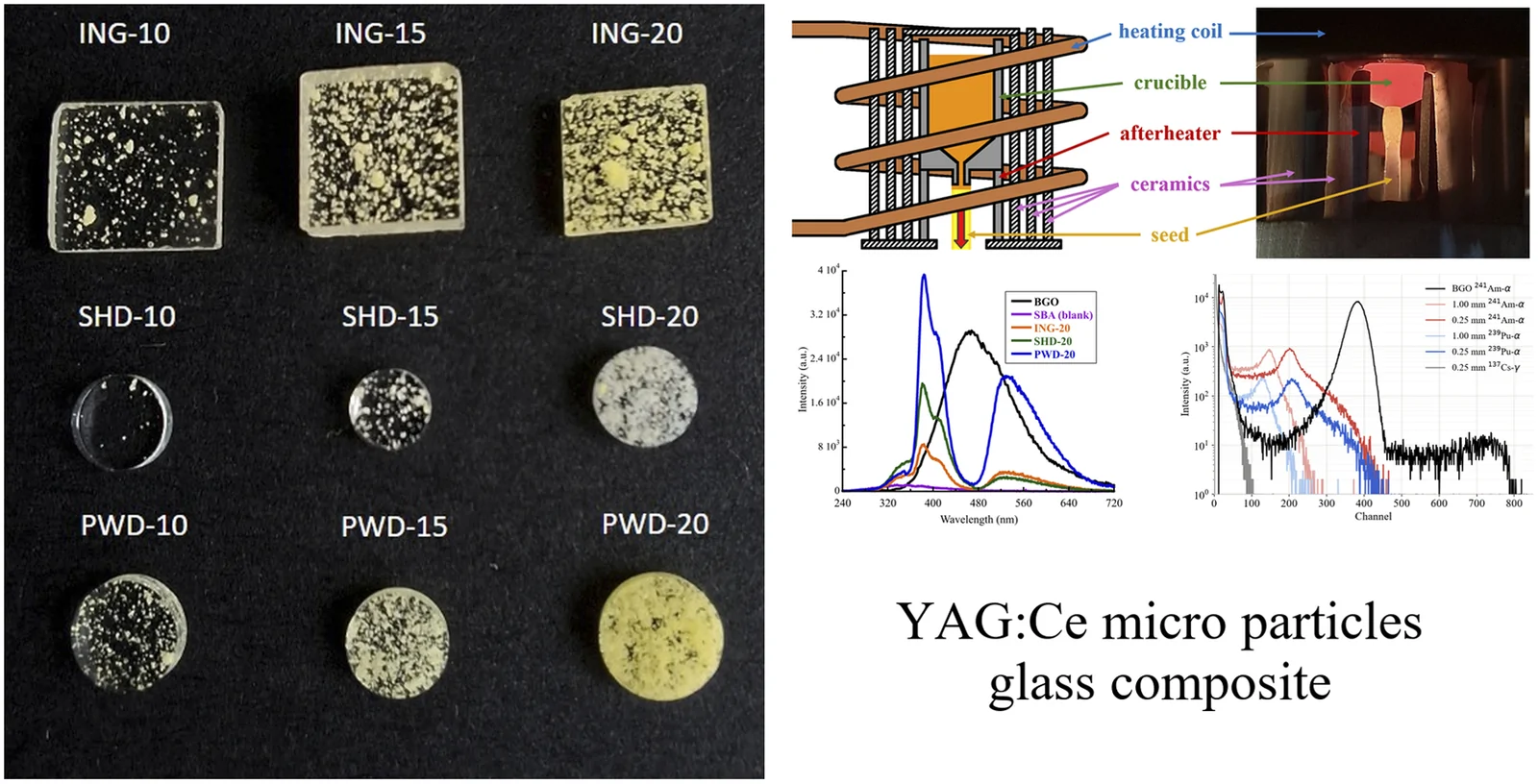

## 1 Introduction

Scintillators play a crucial role in the detection of ionizing radiation across a broad range of applications, including medical imaging,^1^ high-energy physics,^2^ homeland security,^3,4^ and environmental monitoring.^5^ Upon interaction with ionizing radiation, scintillators convert absorbed energy into visible, UV or near infra-red light, enabling signal detection through photodetectors. Scintillators can be fabricated in various forms, including plastics, powders, transparent ceramics, glasses and single crystals. Among these, single crystal scintillators remain the most widely used due to their high light output, excellent energy resolution, and fast, well-defined decay times.^6^ However, the production of high-quality single crystals is very costly and time-consuming.

The growth of single-crystal scintillators commonly takes days or weeks at very high temperatures, often exceeding 1700 °C. Such extreme conditions demand specialized high temperature furnaces, precise thermal control, and chemically inert growth environments. In many cases, iridium crucibles are required due to their chemical stability and resistance to corrosion at elevated temperatures. The cost of iridium has risen dramatically in recent years, significantly increasing the overall production expenses of single crystal scintillators. These factors combined contribute to a continuously increasing price of high-performance scintillation materials.

Given these economic and technological challenges tied to production of single crystals, there is a strong motivation to explore alternative, cost-effective materials for ionizing radiation detection systems. Scintillation glasses are frequently reported, see recent review^7^ where luminescent centers (doped ions) are embedded in a glass host. Their much lower cost compared to single crystals is certainly an advantage, but poor energy transfer to luminescent centers results in comparatively lower light yield. A novel strategy is to introduce scintillating particles into a low-melting-point matrix, such as glass, resulting in a multifunctional composite material. In this concept, the scintillation functionality is retained within dispersed micro- or nano particles, while the glass matrix ensures mechanical stability, optical transparency, and a possibility of shaping, depending on the intended application. In most recent studies, such a material is obtained as glass prepared by standard melt quenching method, which is subsequently annealed in order to introduce nano or micro sized crystals into the system.^8,9^ These materials show very promising properties, such as light yields up to 26 000 pH/MeV.^10^

In this work, we present a modified micro-pulling-down (µ-PD) method^11^ for the preparation of glass-based composite materials containing scintillating particles. The proposed approach enables rapid fabrication of glass rods at low processing temperatures, offering a cost-effective and time-efficient alternative to conventional crystal growth techniques. Due to the steep temperature gradient at the capillary outlet and the elevated melt flow velocity within the capillary channel, the µ-PD process provides favorable conditions for instantaneous solidification and stabilization of the amorphous glass phase. Furthermore, the previous reports revealed that the hydrodynamic stresses and possible cavitation phenomena occurring inside the capillary may promote particle deagglomeration, therefore improving dispersion of the scintillating phase within the matrix.^12,13^ The modification of the µ-PD setup, growth optimization, as well as optical characterization of prepared samples, are described in detail in the following sections.

## 2 Experimental

In this section, we describe the selected materials and provide a detailed description of the preparation procedures and characterization methods used for the composite samples.

### 2.1 Materials

For the experiments, a borosilicate-type glass obtained from a collaborating research group was used and denoted as SBA (or UV-710 in the original study^14^). The glass matrix was prepared using the conventional melt–quenching technique. The glass was formulated on a weight percentage basis with the following nominal composition:53SiO_2_–28B_2_O_3_–1.5Al_2_O_3_–5Li_2_O–5Na_2_O–7.5K_2_O

High-purity (3 N) raw powders were mixed in an agate mortar for approximately 20 to 30 min to ensure batch homogeneity. The homogeneous mixture was then transferred into a platinum crucible and melted in an electric furnace at 1250 °C for 180 min. During the melting process, the melt was stirred three times to ensure homogeneity. The melt was subsequently poured into preheated graphite molds and cooled at a rate of 0.5 °C/min. The resulting ingots were crushed into smaller fragments and milled into a fine powder. The combination of SiO_2_ and B_2_O_3_ provides suitable viscosity, allowing the melt to flow through the capillary while maintaining sufficient stability for shaping. The presence of boron could potentially enable application in thermal neutron detection.^3^

The particular combination of Li_2_O, Na_2_O and K_2_O, and the ratio between them, were not optimized in the present study. This composition was developed and reported previously for an unrelated purpose, as the low-index member of a glass pair for all-solid photonic-crystal fibres,^14^ and was made available to us by the originating group already formulated and in a quantity sufficient for the entire sample series, which had the practical benefit of eliminating matrix batch-to-batch variation as a source of scatter between our samples. It was adopted because two of its properties were already appropriate for the task: the mixed-alkali borosilicate network provides a viscosity–temperature characteristic and a glass-transition range compatible with capillary flow and rapid shaping in the µ-PD geometry, and its total alkali content is comparatively low, which we independently judged favourable for limiting dissolution of the dispersed garnet particles in the melt.

The Y_3_Al_5_O_12_:Ce (YAG:Ce) micro powders, doped with 1 at% Ce, were prepared by a freeze-drying technique. Aqueous slurries were prepared from high-purity oxide powders: Y_2_O_3_ (99.9%), Al_2_O_3_ (99.99%) and CeO_2_ (99.99%). The powder mixture was homogenized in a planetary mill with Dolapix CE 64 as the dispersant, Duramax B-1000 as the binder, and octakis(tetramethylammonium)-T8-silsesquioxane hydrate (Octaanion) as the sintering aid. The well-dispersed slurries were sprayed into liquid nitrogen to obtain granulated precursors, which were freeze-dried (lyophilized) for 10 to 12 h. The resulting granules were calcined in air at 950 °C for 1 h to remove organic additives, followed by solid-state synthesis at 1600 °C for 2 h to promote formation of the YAG:Ce phase.

### 2.2 Experimental setup

Samples in the form of glass rods were prepared using a modified micro-pulling-down (µ-PD) setup, which is traditionally used for rapid and efficient growth of single crystals. The method utilizes a crucible placed inside a so-called hot zone, consisting of structural and shielding ceramic components positioned within an induction coil. This configuration enables operation at temperatures up to approximately 2100 °C. Once the charge melts, the melt flows through a capillary (typically 0.50 mm in diameter) and solidifies shortly after leaving the crucible. Because solidification occurs rapidly, significant thermal stress can develop within the material; therefore, an after heater is commonly used to moderate the temperature gradient (see left part of Fig. 1).

**Fig. 1:**
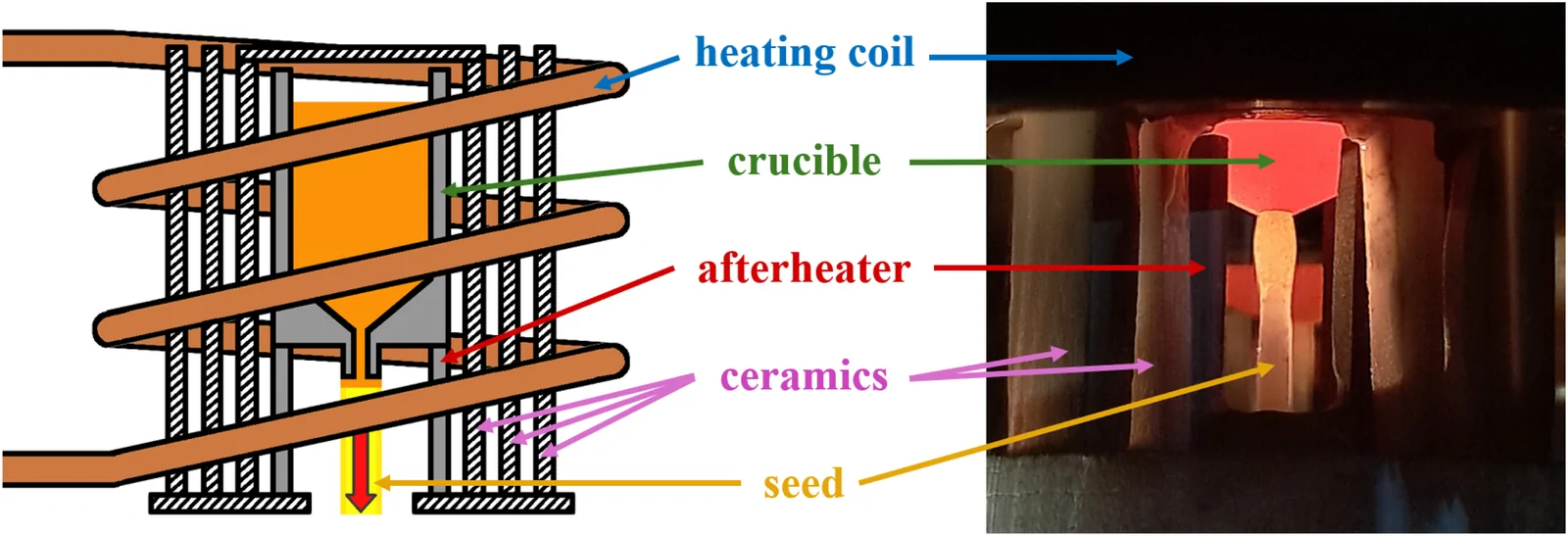
Comparison of traditional design of the µ-PD hot zone (left) and hot zone modified for the preparation of glass composites (right).

Since glasses exhibit different behavior at elevated temperatures compared to crystalline materials, substantial modifications to the setup were required, primarily within the hot zone. The main challenge arises from the fact that glass-forming materials do not solidify instantaneously upon cooling. Instead, they transition into a viscous (supercooled) liquid state, in which they display characteristics of both liquids and solids. This state presents a technological challenge, as the viscous melt can neither be reliably pulled nor flow freely out of the crucible. To minimize the region in which the material remains in this viscous state, the melt must be cooled as rapidly as possible. To promote fast cooling, large windows were cut into both the afterheater and the shielding ceramics below the crucible level, ensuring that the crucible remained thermally shielded while the emerging glass rod was exposed (see right part of Fig. 1). Rapid solidification was further promoted by using a YAG crystal as a seed, which enabled effective heat sink. The tip of the crystal was machined to a sharp point to enable insertion into the crucible, locally undercool the melt, and initiate the pulling process. Multiple standard graphite crucibles with 2 mm capillaries, as well as various die shapes and lengths, were tested. The most stable growth conditions were achieved using a crucible with a conical bottom and no die. To improve thermal insulation of the melt and efficient induction heating, an additional crucible was placed on top of the primary crucible. The modified experimental setup is shown in right part of Fig. 1.

### 2.3 Samples preparation

To assess the effect of the µ-PD process on the optical properties of the composite samples, three distinct series of samples were prepared and subsequently compared. The first series of samples (denoted as ING) was prepared by incorporating YAG:Ce 1 at% micro powders into the molten SBA glass (see Section 2.1). The YAG:Ce powder content varied systematically from 10 to 20 wt%, with a step of 5 wt%, relative to the glass mass (100 g). The total mass of the YAG:Ce micro powders was divided into four equal portions, which were stepwise added to the molten glass while continuously stirring to ensure uniform dispersion of the phosphor particles within the glass matrix. After complete incorporation and homogenization of the YAG:Ce micro powders, the resulting glass-phosphor composite was melt-cast into a preheated graphite mold, transferred into an annealing furnace and cooled to room temperature at a controlled cooling rate of 0.4 °C/min. The solidified glass ingots were then removed from the molds and samples were cut and polished from the central part of each ingot. These samples were prepared mainly to serve as a benchmark for evaluating the influence of the µ-PD process on optical properties of following samples. The second series of samples (denoted as SHD) was prepared by crushing the particle-containing ingots into smaller shards, which were subsequently used as a source material for a µ-PD experiment. The idea behind this series of samples was to observe if the particle distribution can be improved in a system which already underwent thermal treatment.

The third series of samples (denoted as PWD) was produced by directly mixing a fine SBA glass powder with YAG:Ce micro particles. The weight of precursor powders was set to 0.9 g, as the flow of glass through the capillary depends not only on the melt temperature but also on the hydrostatic pressure of the melt above. The precursor glass was initially milled into coarse powder using a FRITSCH Ball Vibratory Micro Mill PULVERISETTE 0, with a corundum jar and milling ball. The material was then further pulverized to a fine powder using a mortar and pestle. After weighing the mixture of glass and micro particles, it was homogenized by mechanical agitation in a small vial for several minutes, followed by a second round of grinding using a mortar and pestle. The powder was then placed into the crucible, and the hot zone was assembled in the µ-PD setup. After assembling the hot zone, the growth chamber was enclosed with a cylindrical quartz glass cover and evacuated to a pressure below 10 Pa using a rotary vane pump. While maintaining the vacuum, the furnace power was increased to the growth condition (22 to 24% of maximum power) and held for 15 to 20 min to remove adsorbed gases, which would otherwise form small bubbles within the glass samples. The vacuum pump was then turned off, and the growth chamber was immediately pressurized to approximately 85 kPa with inert gas and sealed. Both argon (6 N purity) and nitrogen (4 N) were deemed satisfactory, and nitrogen was used for subsequent experiments due to its lower cost.

Seed touch was established by inserting the YAG seed crystal approximately 2 mm into the crucible capillary. Using a pulling speed of 1.0 to 1.5 mm/min and a possible power increase of 2 to 3%, we achieved the desired glass rod diameter at a distance of roughly 6 mm from the crucible capillary, corresponding to the point where the glass reaches its glass-transition temperature and becomes non-ductile. Growth continued at this pulling rate and power setting, with fine adjustments (below 1% in power and 0.2 mm/min in pulling speed) to maintain a stable diameter, and a slight increase (1 to 3%) in power toward the end of the run to compensate for the decrease in hydrostatic pressure of the melt in the crucible. Using this approach we were able to prepare glass rods containing YAG:Ce micro particles (MPs) that were free of macroscopic bubbles detectable by optical microscopy over approximately the first 80% of the rod length. Every rod grown for this study was inspected in this way. Using 0.9 g of precursor powders enabled us to pull rods up to 10 cm long, which is the limit of our apparatus. The diameter of the rods could be adjusted by controlling the coil power output and the pulling speed, enabling us to reach diameters in the range 3–7 mm while maintaining a uniform cross-sectional shape. Photographs of the prepared rods are shown in Fig. 2 (left). The full list of samples cut from the rods and their labels is given in Table 1 and Fig. 2 (right).

**Fig. 2:**
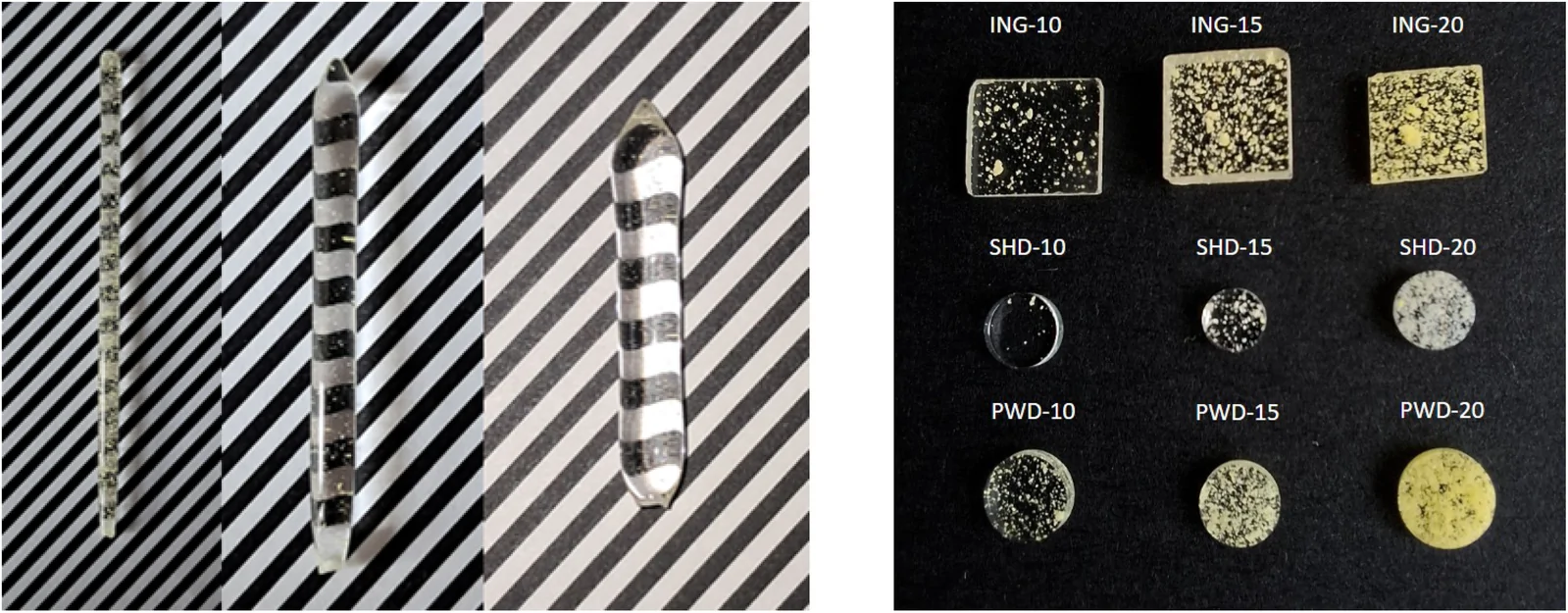
Left: glass composite rods pulled to different user-selected diameters; all photographs were taken against paper with 3 mm stripes, which serves as the scale. Right: photograph of the cut and polished samples, arranged as in Table 1 (rows: ING, SHD, PWD; columns: 10 wt%, 15 wt% and 20 wt% YAG:Ce).

**Table 1:** List of samples and their respective contents of YAG:Ce micro particles (MPs) expressed in wt%

Method of preparation	10 wt%	15 wt%	20 wt%
Melt quenching (ingots)	ING-10	ING-15	ING-20
µ-PD (from shards)	SHD-10	SHD-15	SHD-20
µ -PD (from powder)	PWD-10	PWD-15	PWD-20

The terminal part of each rod, roughly the last 20% of its length, consistently showed a visibly reduced YAG:Ce particle concentration, which we attribute to progressive depletion of the particle fraction in the residual melt as the run proceeds. This section was discarded, and all samples reported in this work were cut from the preceding, visually homogeneous part of the rod.

### 2.4 Characterization techniques

X-ray diffraction (XRD) patterns were measured using a Rigaku MiniFlex diffractometer with Cu K*α* radiation (*λ* = 1.5406 Å), operated at 40 kV and 15 mA. Data were collected over the 2*θ* range of 10° to 80° in continuous scan mode with a scan speed of 2.5°/min.

Absorption spectra were measured with a Shimadzu 3101 PC ultraviolet–visible–near-infrared (UV/VIS/NIR) spectrophotometer over the range 190 to 600 nm.

Radioluminescence (RL) spectra were measured with a custom-made 5000M spectrofluorometer (Horiba Jobin Yvon, Wildwood, MA, USA) using a W-anode X-ray tube (40 kV, 15 mA, Seifert) as the excitation source. The detection part of the setup comprised a single-grating monochromator and a TBX-04 photon-counting detector (Horiba, IBH). The measured spectra were corrected for the spectral dependence of the detection sensitivity. For every RL spectrum, a BGO (Bi_4_Ge_3_O_12_) reference single crystal was measured under identical geometrical conditions in order to obtain quantitative intensity information; all RL intensities in this work are expressed as a percentage of that reference.

This setup was also used for the measurements of photoluminescence excitation (PLE) and emission (PL) spectra, as well as photoluminescence decays. For these measurements a steady state laser driven xenon lamp (Energetiq, a Hamamatsu Company) and nanosecond nanoLED pulsed light sources in time-correlated single-photon counting mode (Horiba Scientific) were used as the excitation sources, respectively.

Scintillation decay curves were measured with a Hamamatsu R375 photomultiplier (sensitivity range 160 to 850 nm, rise time 9 ns) and a Tektronix TDS3052C digital storage oscilloscope. The excitation source was 662 keV *γ*-radiation from a ^137^Cs radioisotope source. The final scintillation decay curve is an average of approximately a thousand individual decay curves.

The PL and PLE spectra are corrected for the experimental distortion. The convolution procedure was applied to the decay curves to determine true decay times (SpectraSolve software package, Ames Photonics).

The decay curves are approximated using least-square method by the sum of *n* exponentials in the following form:
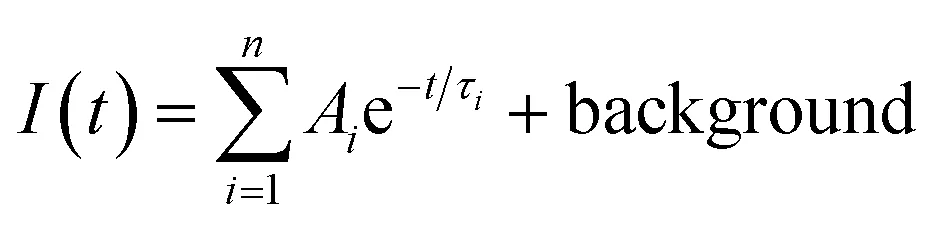
where *A*_*i*_ and *τ*_*i*_ are the amplitude and decay time of the *i*th component, respectively. Relative error of time components obtained is <1.5% for the fast components and <3% for the slow components.

The portion of light released by the *i*th component, which is commonly called a light sum (LS_*i*_, expressed in %), can be calculated as:
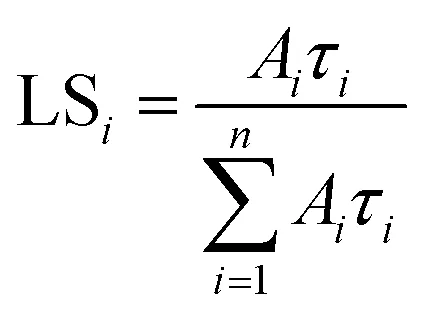


Pulse-height spectra were recorded under ^137^Cs *γ*-ray, ^239^Pu and ^241^Am *α*-particle excitation using a hybrid photomultiplier (HPMT, model PP0475B), an ORTEC 672 spectroscopy amplifier (shaping time 1 µs), and an ORTEC ASPEC-927 multichannel analyzer. PTFE tape was used as a reflector for γ excitation, and optical coupling between the sample and the photomultiplier was provided by DOWSIL Q2-3067 optical couplant.

## 3 Results and discussion

In this section, we describe the phase and optical characteristics of the prepared samples. The main focus is a comparison between the three sample series (see Section 2.3) in order to assess the effect of the modified µ-PD technique on the properties of the glass composites.

### 3.1 Phase characterization

A comparison of the XRD patterns of samples containing 20 wt% of YAG:Ce MPs is shown in Fig. 3. In sample ING-20, the garnet phase is observed, which we attribute to the micro particles, alongside the YBO_3_ phase. Following the additional thermal treatment during µ-PD growth, the YBO_3_ phase becomes more prominent in SHD-20. The sample PWD-20 contains both phases, although the relative peak intensities indicate that the YAG phase remains dominant.

**Fig. 3:**
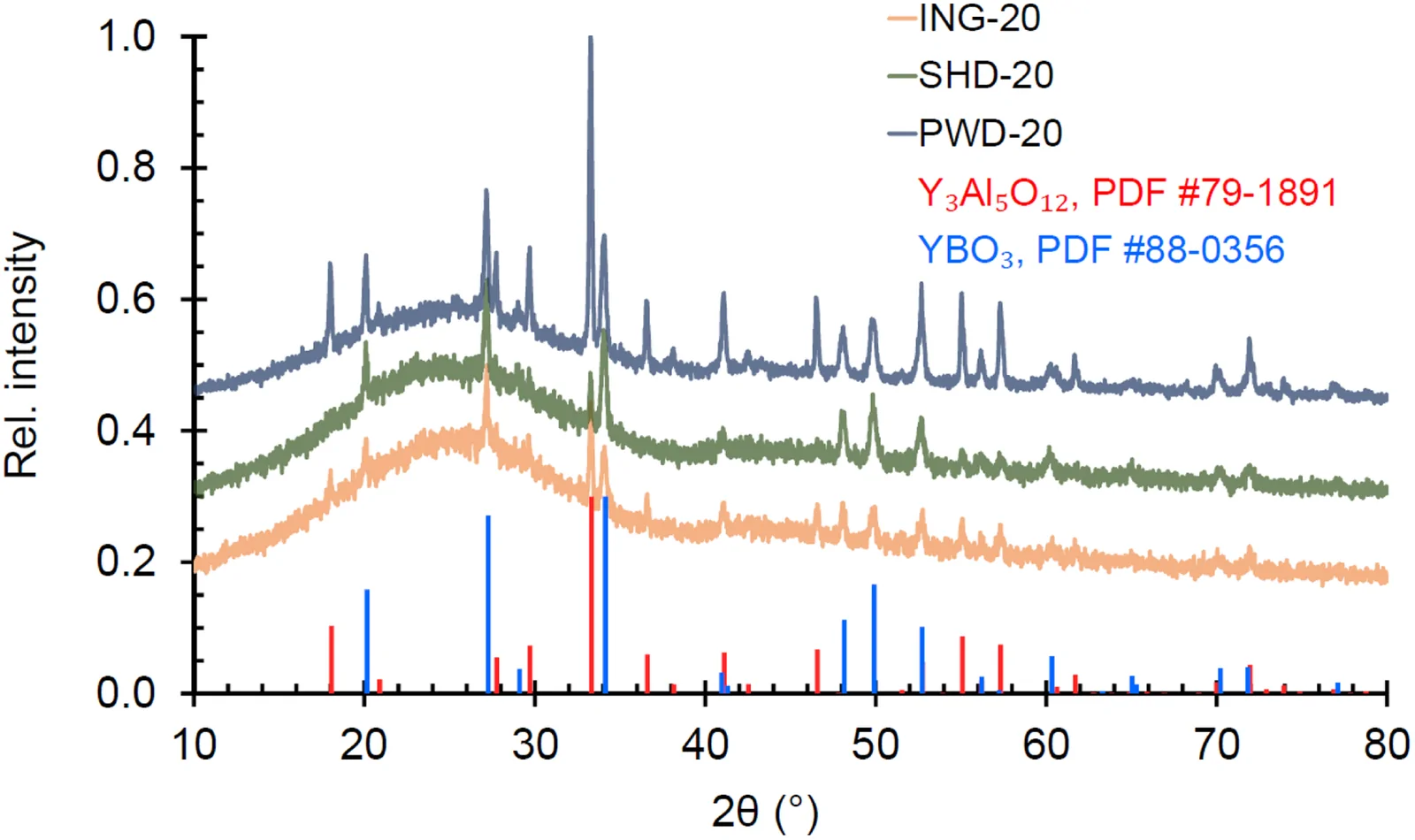
XRD patterns of the three samples containing 20 wt% YAG:Ce micro particles (ING-20, SHD-20, PWD-20), showing the garnet (YAG) and YBO_3_ reflections. Cu K*α* radiation, *λ* = 1.5406 Å.

The formation of YBO_3_ can be described as a heterogeneous reaction at the interface between the solid phase and the highly viscous glassy phase. The kinetics of this process are therefore expected to be strongly influenced by the diffusion of the reaction products away from the interface and into the glassy phase. This is reflected in the XRD patterns and in the subsequent measurements, which show that the SHD samples subjected to multiple heat treatments contain comparatively larger amounts of the YBO_3_ phase. It is also important to note that the mobility of the reaction products is strongly limited within the highly viscous glassy phase. Consequently, recrystallization may occur at near proximity to the surface of the YAG particles, forming a layer that effectively shields the particles from further dissolution. Based on this mechanism, it can be assumed that the YAG micro particles are unlikely to be completely dissolved within the time required for the µ-PD experiment, which is about an hour at peak temperature.

### 3.2 Optical characterization

Absorption spectra of all samples are characterized by good transparency in the visible region while showing a set of absorption bands in the range 220 to 340 nm (see Fig. 4a). This absorption can be ascribed to the Ce^3+^ 4f → 5d transitions in spontaneously created microphase of YBO_3_ present in the samples^15^ (see more details below).

**Fig. 4:**
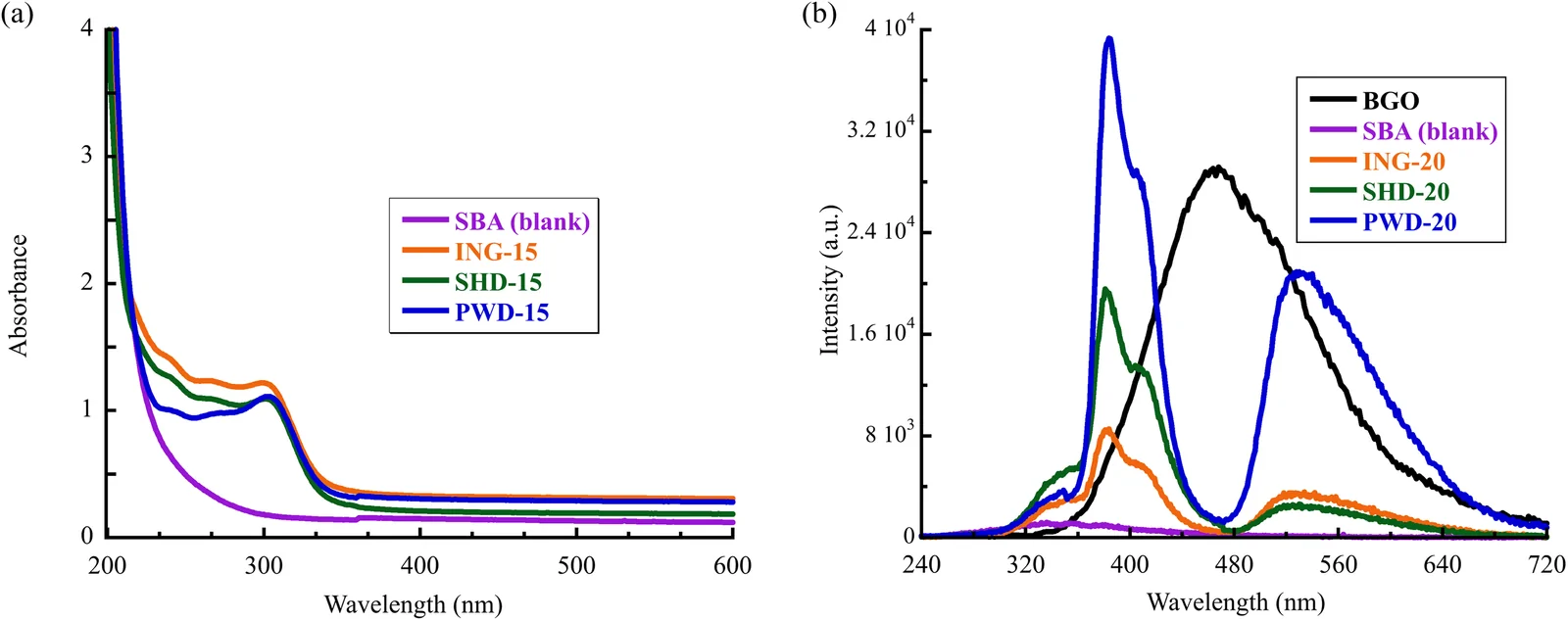
Absorption and radioluminescence spectra of the glass composite samples. RL spectra were excited with the W-anode X-ray tube (40 kV, 15 mA) under identical geometrical conditions for all samples and for the BGO reference. Each curve is a single-specimen acquisition (*n* = 1). (a) Absorption spectra of the samples containing 15 wt% YAG:Ce. (b) Radioluminescence spectra of the samples containing 20 wt% YAG:Ce, together with the BGO reference and a particle-free SBA blank

Radioluminescence (RL) spectra show multiple intense bands (see Fig. 4b), that can be separated into two sections. First, the broad emission band with maxima at around 530 nm is typical for 5d → 4f transition of Ce^3+^ ion in a garnet lattice.^16^ Moving on to shorter wavelengths, we can notice a set of band emissions with maxima at 410, 380 and 355 nm. Maxima at 410 and 380 nm can be attributed to luminescence of Ce^3+^ positioned within crystalline YBO_3_, which has been described well in available literature.^15,17^ This furthermore confirms a partial dissolution of YAG:Ce particles during all preparation processes. Lastly, the broader emission arising at around 355 nm is an intrinsic luminescence of the SBA matrix. When comparing samples of equal YAG:Ce concentration (see Fig. 4b), both 530 nm emission of YAG:Ce and emission bands related to YBO_3_ are the most intense for samples prepared from powders. It is evident, that the rising intensity of YBO_3_-related emission is not caused by higher rate of YAG:Ce dissolution. It can therefore be assumed that the modified µ-PD approach provides the best particle distribution within the glass matrix, partially suppressing concentration quenching, self-absorption, or both. On the other hand, the increase of intensity of emission related to YBO_3_ in SHD-20 sample is due to higher degree of YAG:Ce dissolution, which is also represented by the decrease of 530 nm emission intensity. This observation is in agreement with the XRD measurements.

Integral RL intensities of all emission bands were calculated and compared, their values expressed as % of the BGO reference sample are listed in Table 2. The values have a relative error of about 5% due to the RL spectra not being perfectly reproducible (sample placement, holder orientation *etc.*) and should therefore be understood as a preliminary estimation of the scintillation performance. The preparation of the samples from shards slightly increased total integral intensities, but the increase of YBO_3_:Ce emission (270 to 470 nm) is accompanied by a decrease of YAG:Ce emission (470 to 720 nm). On the other hand, the preparation of the samples from powder exhibits superior total intensity (270 to 720 nm) as well as a significant increase in the garnet emission alone. These values further highlight the impact of preparation methodology.

**Table 2:** Integral radioluminescence intensities of the YBO_3_:Ce (270 to 470 nm) and YAG:Ce (470 to 720 nm) emission components of the samples containing 20 wt% YAG:Ce, expressed as % of the BGO standard

Integral intensities compared to BGO (%)				
Sample	YBO_3_:Ce	YAG:Ce	YAG/YBO_3_	Total
ING-20	12	8	68	21
SHD-20	27	6	23	33
PWD-20	44	50	114	94

To better understand the luminescence characteristics of the composite samples, the PLE and PL spectra were measured. All the excitation spectra measured at *λ*_em_ = 530 nm show an intense band with maxima at 460 nm alongside two less intense bands at 335 and 360 nm. While the excitation bands at 460 and 335 nm can be ascribed to Ce^3+^ 4f → 5d^1^ and 4f → 5d^2^ transitions, respectively, in the YAG structure, the 360 nm band originates elsewhere (see Fig. 8a). A possible origin of this band was revealed by measuring excitation spectra for *λ*_em_ = 410 nm, which is the emission ascribed to YBO_3_:Ce. Excitation spectra for *λ*_em_ = 410 nm show two bands at 320 and 360 nm which change in intensity depending on YAG:Ce concentration (see Fig. 8b). Since the borate emission at 410 nm overlaps with 4f → 5d^1^ excitation band of YAG:Ce, the conditions for energy transfer from YBO_3_:Ce to YAG:Ce are met. In order to confirm or deny this process occurring in our samples, emission spectra for *λ*_ex_ = 365 nm were measured. Fig. 8c shows a clear overlap in the emission spectra for *λ*_ex_ = 365 nm and excitation spectra for *λ*_em_ = 530 nm. To finally confirm the energy transfer process, photoluminescence decay was measured at *λ*_ex_ = 362 nm and *λ*_em_ = 530 nm. The decay curve in Fig. 8d is characterized by a rise time of 28 ns and a decay time of 65 ns, which confirms a radiative energy transfer from YBO_3_:Ce to YAG:Ce, as the rise time is close to the decay time of the 410 nm emission of YBO_3_:Ce, see below.

Photoluminescence decays were also measured separately for YBO_3_:Ce and YAG:Ce. The decay curve measured at *λ*_ex_ = 455 nm and *λ*_em_ = 530 nm is typical for Ce^3+^ within a garnet lattice, showing a fast component of 54 ns and a slow component of 249 ns.^18^ However, the light sum of this slow component is only 4.4%. The decay curve for YBO_3_:Ce (*λ*_ex_ = 365 nm, *λ*_em_ = 410 nm) is characterized by two fast components of about 30 ns and 50 ns respectively. The ratio of light sums between these two components is 75 to 25% in most cases. We can also notice the presence of slow component, which could not be properly fitted due to a very low signal within the 350 to 800 ns time window (see Fig. 9b). The decay time of this component is estimated to be around 600 ns (see scintillation decays below). We ascribe this longer component to the intrinsic emission of the glass matrix. Some part of this component could be due to the onset of the Ce^3+^ excited state ionization of YBO_3_.^19^

All scintillation decay curves under excitation by *γ*-ray quanta of 662 keV energy from ^137^Cs radioisotope show a fast component of around 45 ns with light sums up to 78%. The second component yielding roughly 22% of the detected light typically ranges from 550 to 650 ns. The fast component is ascribed to prompt YBO_3_:Ce and YAG:Ce emissions, while the slow light stems from the intrinsic luminescence of the SBA matrix or from charge-carrier trapping in the transfer stage of the scintillation mechanism.

### 3.3 Pulse-height spectra, afterglow

To estimate the light yield (LY) and energy resolution (ER), pulse height spectra were measured for the PWD-20 rod. At first, a standard 1 mm sample was measured under 662 keV *γ*-photons of ^137^Cs but no peak of total absorption was present in the spectra. The absence of total absorption peak can be caused by a combination of two factors. Firstly, the effective atomic number and density are probably too low in order for the 662 keV photons to fully absorb. Secondly, a reabsorption of the emitted light by the sample itself may occur. To suppress these effects, a 0.25 mm sample of PWD-20 was prepared and exposed to *α*-particles of ^241^Am and ^239^Pu radioisotopes. These measurements provided spectra with well-resolved photo peaks that were fitted by a Gaussian function. In order to see the influence of sample thickness on the pulse-height spectra measurements, the original 1 mm sample was also measured under the same *α*-particle sources. Fig. 5a shows the measured pulse height spectra, the calculated values of extracted LY, ER as well as measurement specifications can be seen in Table 3.

**Fig. 5:**
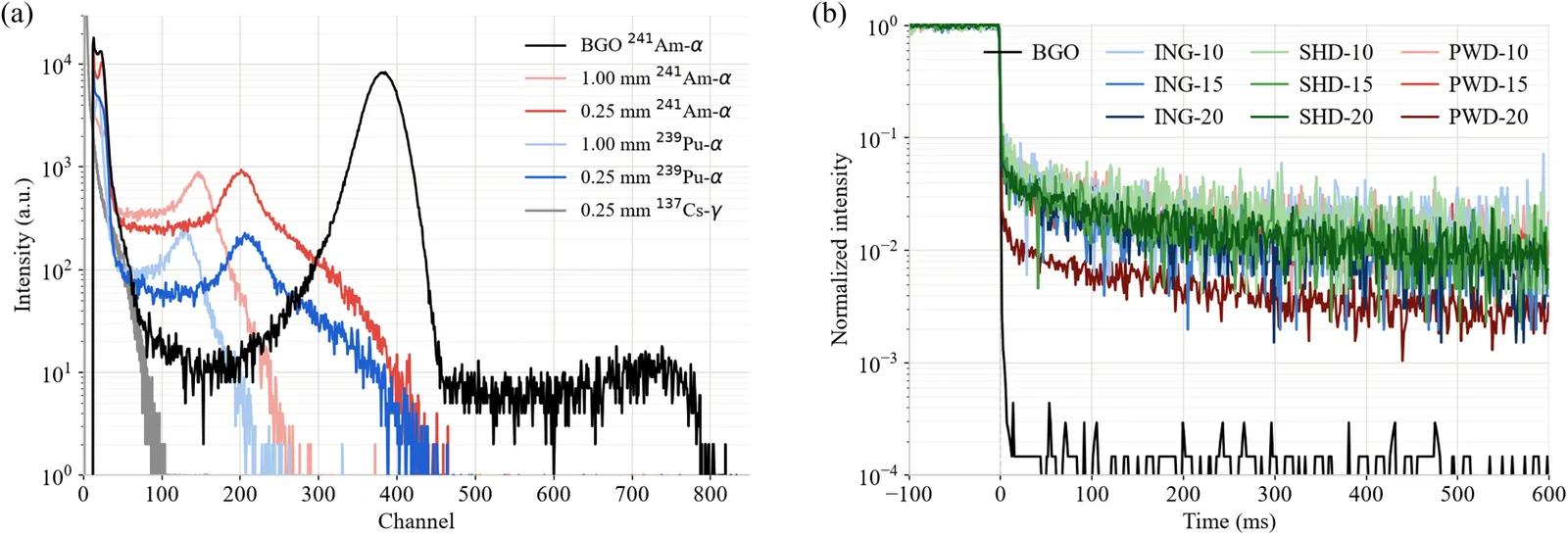
Pulse-height spectra and afterglow of the samples. For pulse-height spectra, samples were excited by ^137^Cs *γ*-ray, ^239^Pu and ^241^Am *α*-particle radioisotopes under identical geometrical conditions. For afterglow curves, cutoff of W-anode X-ray tube (40 kV, 15 mA) was used under identical geometrical conditions for all samples and for the BGO reference. (a) Pulse-height spectra of PWD-20 and reference BGO. (b) Afterglow curves measured for all samples from the sample set.

**Table 3:** Scintillation properties of 1 mm or 0.25 mm thick PWD-20 samples as well as the BGO standard. The excitation sources were ^241^Am (*α*, 5486 keV), ^239^Pu (*α*, 5157 keV) and ^137^Cs (*γ*, 662 keV)

Sample	Excitation source	LY (pH/MeV)	ER (%)	Thickness (mm)
PWD-20	^241^Am	420(20)	23(1)	1.00
PWD-20	^241^Am	570(30)	19(1)	0.25
PWD-20	^239^Pu	370(20)	24(1)	1.00
PWD-20	^239^Pu	630(30)	21(1)	0.25
PWD-20	^137^Cs	N/A	N/A	0.25
BGO	^241^Am	1180(60)	12(1)	1.00

Pulse-height spectrum measured under 662 keV *γ*-photons of ^137^Cs did not result in a well resolved photo peak even after using the 0.25 mm sample and LY/ER could not be calculated for this energy. The 1 mm sample shows LY of 370 and 420 pH/MeV under ^239^Pu and ^241^Am excitation, respectively. The LY value improves significantly for the 0.25 mm sample, reaching 630 pH/MeV under ^239^Pu excitation. This result confirms the hypothesis of the emitted light being partially reabsorbed by the material. Comparing the LY values to other similar materials is not trivial due to the fact that most works report the LY values recorded under the 662 keV *γ*-photons of ^137^Cs. Chewpraditkul *et al.* have reported a LY of 910 pH/MeV in a Gd_2_O_3_ rich Ce^3+^-doped silica glass,^20^ Ce^3+^-doped phosphate glass was reported to show a LY of 2100 pH/MeV.^21^ Another research group has reported a robust Ce^3+^-activated glass nano-composite with LY of about 853 pH/MeV and energy resolution of 22.27%.^9^ The LY recorded under *α*-particle excitation is typically lower than that measured under *γ*-ray excitation due to the higher ionization density of particles, with the magnitude of the quenching being strongly dependent on the scintillator material and particle energy.

Afterglow of all samples was measured and is presented in Fig. 5b. We can see that behavior of all samples is almost identical, dropping to 1/10 almost immediately and approaching two orders of magnitude drop at 500 ms. Performance of the PWD-20 sample is the outlier, approaching two orders of magnitude drop within tens of ms and approaching 2.5 orders of magnitude at 500 ms.

### 3.4 Reproducibility of the growth process and optical properties

As we present very promising results in the previous paragraphs, it is appropriate to confirm the reproducibility of both the growth process and properties of the as-grown rods. Fig. 6 shows a series of µ-PD grown rods with highlighted places, where diameters were measured by micrometer. It is demonstrated that it is possible to grow glass rods with stable diameter, changing less than 0.4 mm along the length of the rod using the µ-PD method. It is important to mention, that repeatability of the results is very sensitive to operator experience. Industrialization of this process would ideally include automatic diameter control and a continuous supply of the precursor powder in order to keep the diameter stable.

**Fig. 6:**
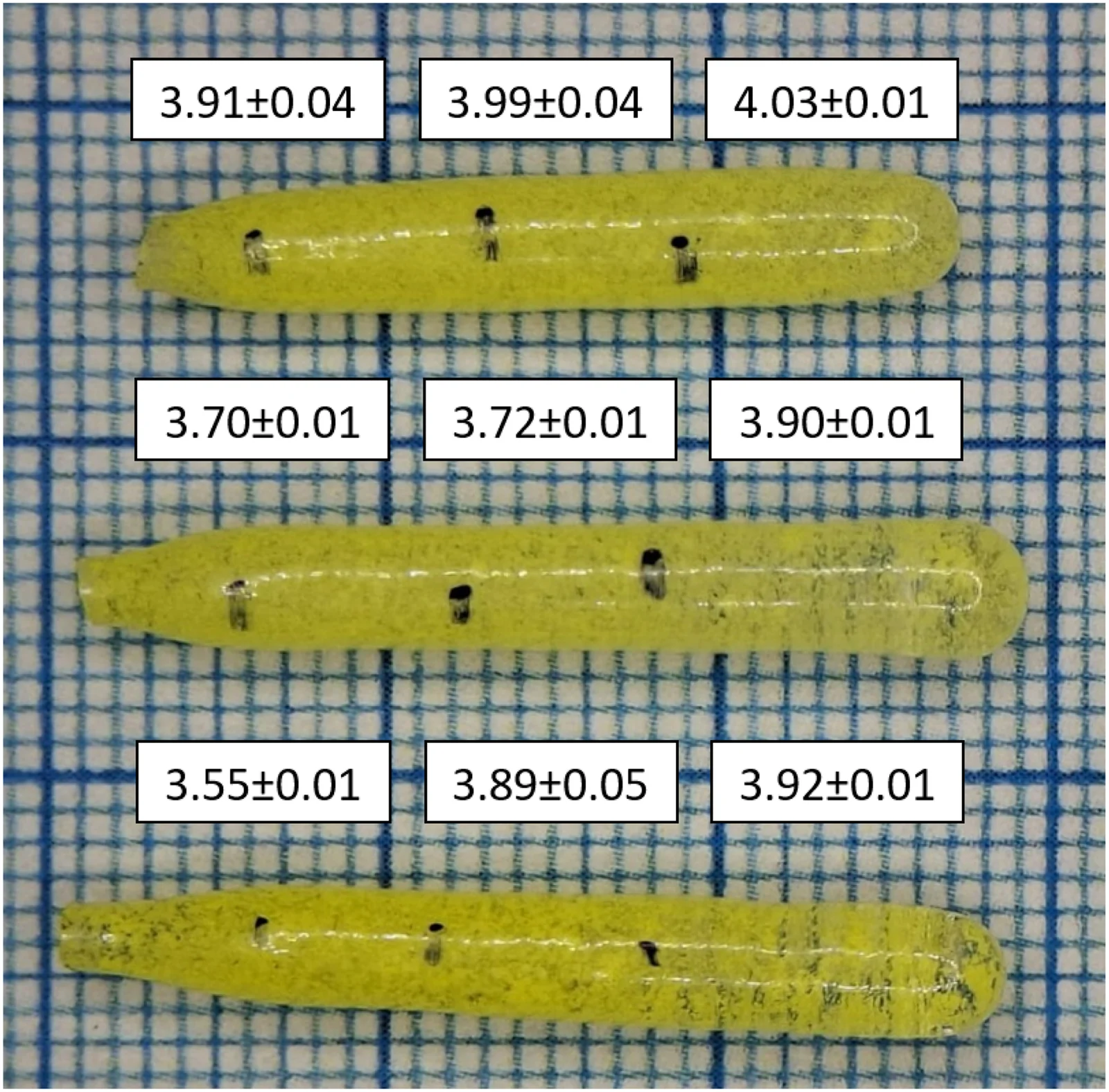
A demonstrative series of glass rods grown by the µ-PD method, showing good reproducibility and stable diameter; the annotated values are the diameters measured by micrometer at the marked positions, in mm, and serve as the scale reference for the photograph.

The homogeneity of optical properties was tested by multiple-site RL measurements. Two samples of 1 mm thickness were cut out and polished from the end (PWD-15-1) and the start (PWD-15-2) of the PWD-15 rod. From the middle section a 3 mm thick cylinder was also cut and polished. This piece was measured on two different spots – the polished face surface (PWD-15-3 (face)) and unpolished side (PWD-15-3 (side)) of the cylinder. Fig. 7 shows the detailed comparison of the radioluminescence spectra of said samples. The spectra are unanimous in shape and their integral RL intensities vary by ±7.5%. This deviation can be caused by both the relative uncertainty of the RL measurement and/or the fact that not all parts of the rod are exactly the same. The latter is highlighted on the drop of intensity of the garnet phase in sample PWD-15-1 (see Fig. 7c) which also shows lower total integral RL intensity comparing to the middle and end section of the rod. The decrease of YAG:Ce intensity at the end of the rod (see Fig. 7c) is caused by gradual depletion of the micro particles during the growth, which is also discussed in Section 2.3. Simultaneously, this part of the melt spends the most time within the hot crucible, explaining the increased intensity of the YBO_3_:Ce band (see Fig. 7a). This deficiency could, once again, be improved by a continuous supply of precursor powder into the crucible. Recycling of the end part as source material for following growths could also be an option and is a common practice in the crystal growth industry.

**Fig. 7:**
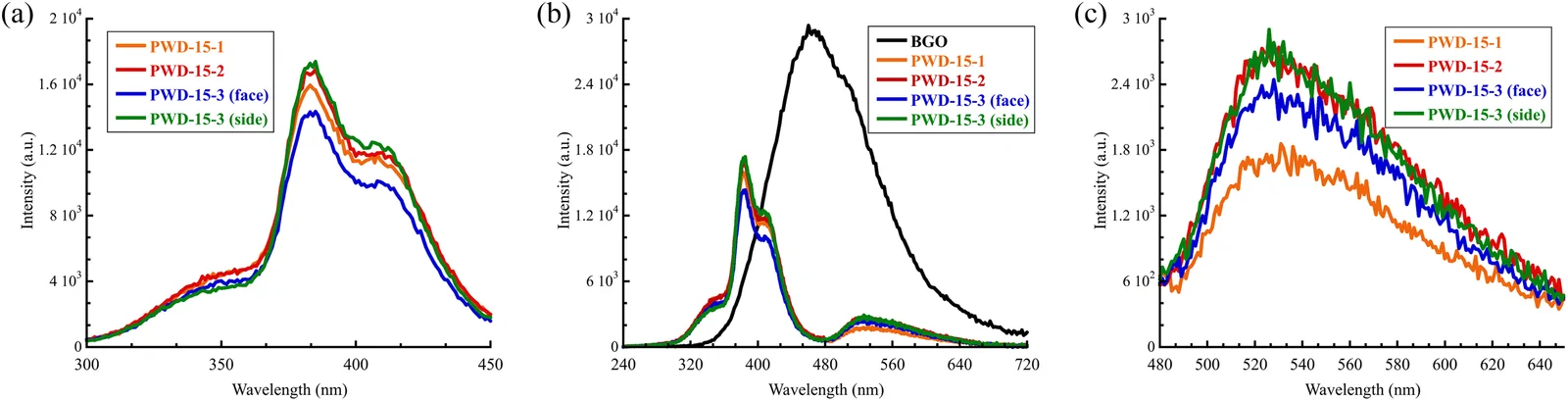
Comparison of RL spectra of the four samples cut from different positions along a single PWD-15 rod (PWD-15-1, PWD-15-2, PWD-15-3 (face), PWD-15-3 (side); see Section 3.4), used to assess the homogeneity of the optical properties along the rod. Each sample is a single specimen (*n* = 1). Spectra were excited with the W-anode X-ray tube (40 kV, 15 mA) under identical geometrical conditions. (a) RL spectra of the four samples prepared from the PWD-15 rod in the range 300 to 450 nm. (b) RL spectra of the four samples prepared from the PWD-15 rod over the full wavelength range. (c) RL spectra of the four samples prepared from the PWD-15 rod in the range 480 to 650 nm.

**Fig. 8:**
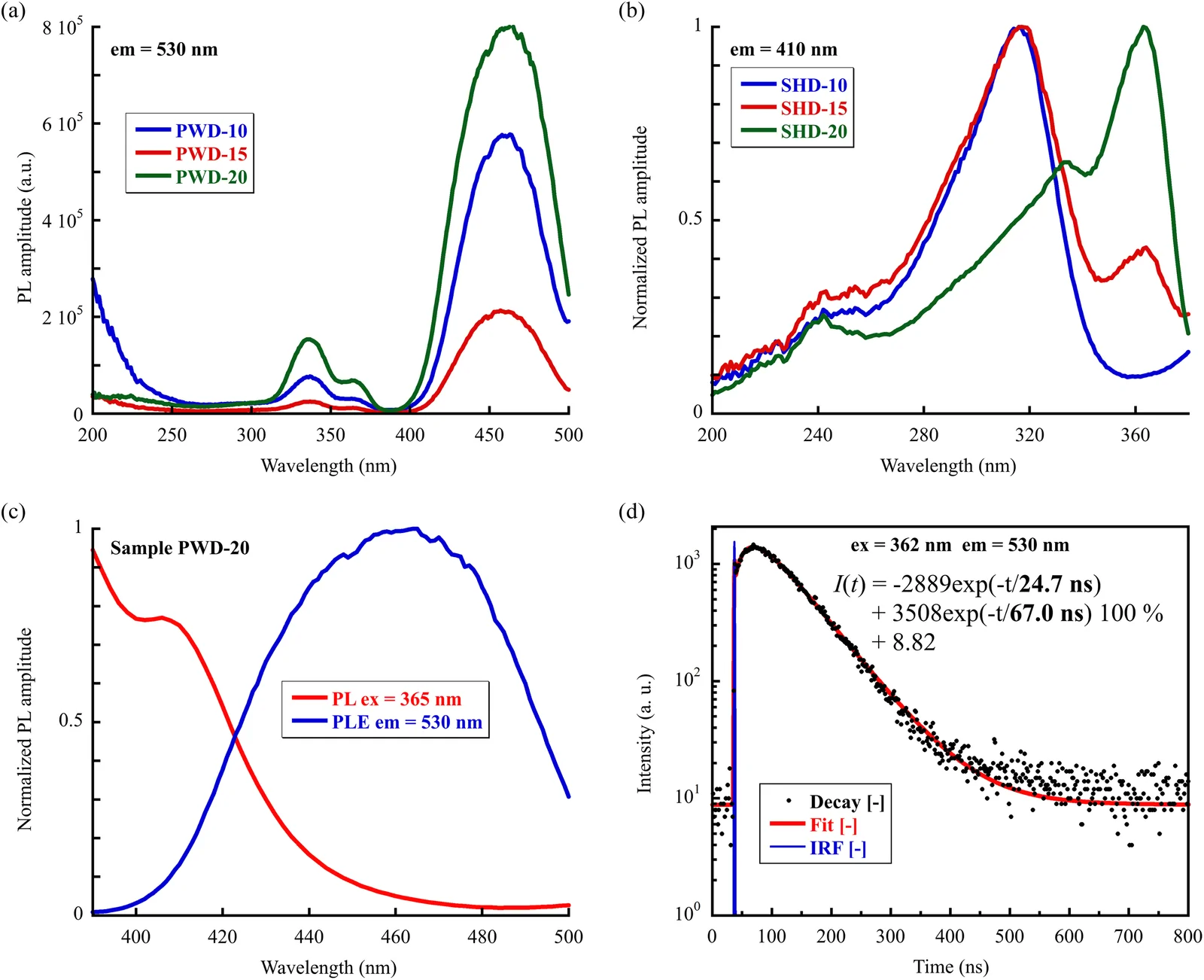
Photoluminescence measurements of PWD and SHD samples (single specimens, *n* = 1) highlighting the energy transfer process between YBO_3_:Ce and YAG:Ce; excitation and emission wavelengths for each panel are given in the corresponding sub-caption.

**Fig. 9:**
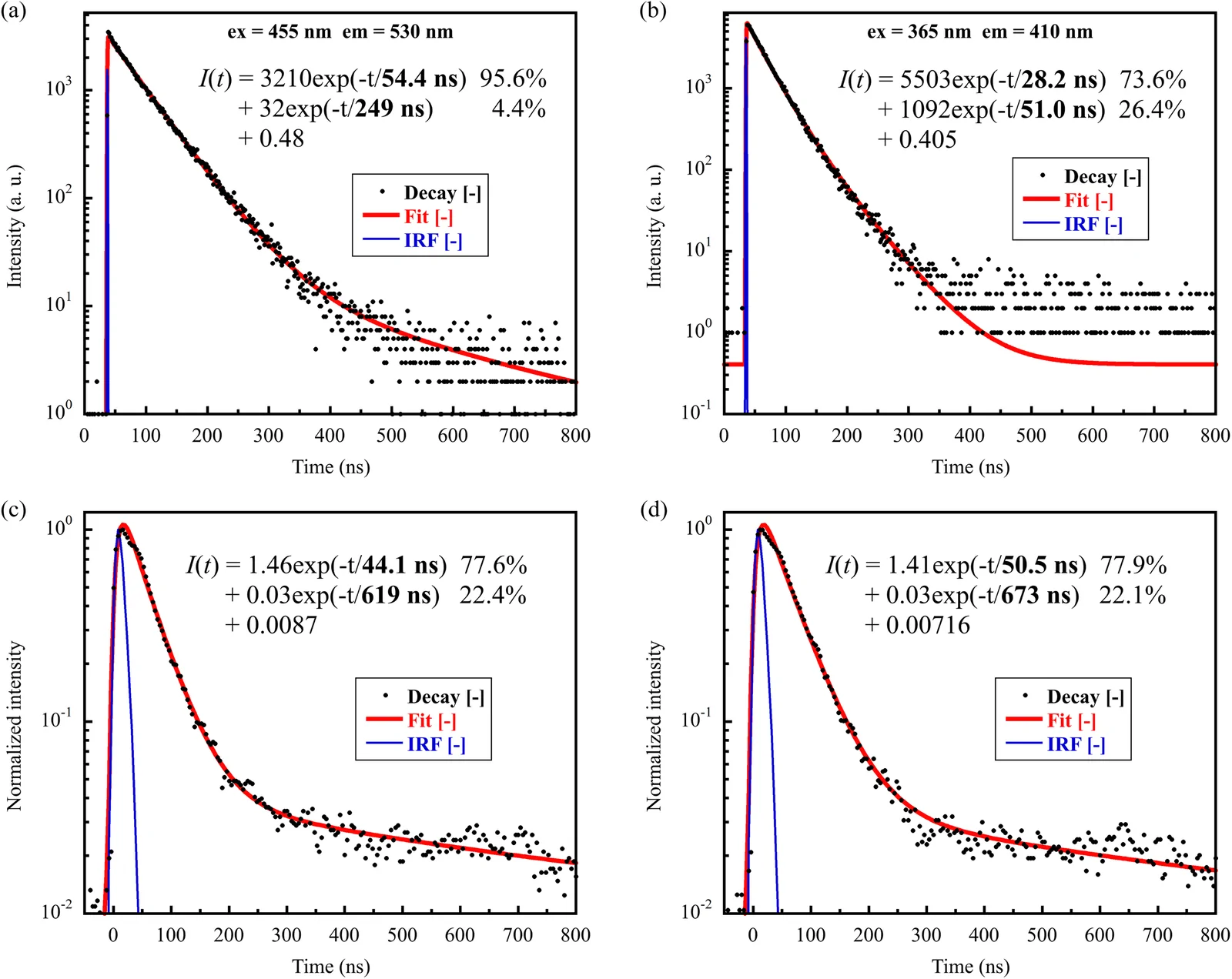
Examples of photoluminescence and scintillation decay curves of PWD samples (single specimens, *n* = 1); excitation/emission conditions for each panel are given in the corresponding sub-caption.

## 4 Conclusions

The preparation of glass–composite rods by modification of the hot zone of the micro-pulling-down apparatus was optimized, enabling the fabrication of samples with controlled diameters in the range of 3–7 mm. Phase analysis confirmed the presence of YAG and YBO_3_ crystalline phases dispersed within the glass matrix, while the corresponding Ce^3+^ excitation and emission spectra confirmed the presence of optically active Ce^3+^ centers.

The approach based on finely ground YAG:Ce micro particles mixed with milled glass proved superior to both melt-quenching and micro-pulling-down using pre-formed glass shards. The radioluminescence of sample PWD-20 reached an integrated RL efficiency corresponding to 94% of that of the BGO reference, compared with only 21% for the reference sample ING-20. All prepared samples exhibited scintillation decay dominated by a fast component, with more than 75% of the emitted light contained within approximately 50 ns. Pulse-height spectra of PWD-20 samples show a well resolved peak of total absorption under ^241^Am (*α*, 5486 keV) and ^239^Pu (*α*, 5157 keV) excitation. The 0.25 mm thick PWD-20 sample shows a LY of 630 pH/MeV and ER of 21% under ^239^Pu excitation. Standard measurements under ^137^Cs (*γ*, 662 keV) excitation did not show any peak of total absorption.

These results demonstrate that glass–composite scintillators based on YAG:Ce micro particles can provide a viable combination of relatively high scintillation output and fast response. The flexibility of the composite approach also provides a pathway toward systematic optimization of glass–micro particle combinations for specific applications. In particular, the approach could enable the efficient utilization of waste material generated during Czochralski growth of single crystals by converting otherwise unusable material into functional scintillating micro particles. The use of borosilicate glass matrices may additionally enable thermal-neutron detection owing to their boron content, while the good shapeability of the material allows the fabrication of complex geometries and large-volume detectors.

The main limitations identified in the present study are related to the maximum rod size achievable with the specific experimental setup and to the decrease in micro particle concentration toward the end of the rods. The latter renders approximately the final 20% of the rod unsuitable for sample preparation. This limitation primarily affects the growth yield rather than the scintillation performance itself and is not uncommon in the preparation of composite or crystalline scintillator materials. The demonstrated process is nevertheless not fundamentally limited to the dimensions achieved in the present study, as larger-scale micro-pulling-down systems with continuous feeding are routinely available.

The present work establishes a proof of concept and provides a basis for several follow-up studies. Further characterization of the prepared composites using SEM/EDX, micro-cathodoluminescence, and XPS is currently being pursued. In parallel, systematic studies of different combinations of glass matrices and scintillating micro particles are underway. Establishing cooperation with industrial partners could further enable the practical utilization of waste material from Czochralski-grown single crystals. For further optimization of the present system, a quantitative study of the kinetics of YBO_3_ formation would be particularly valuable, as it could provide a better understanding and control of micro particle dissolution during processing.

An important advantage of the approach demonstrated in this work is that the glass matrix and scintillating particles can be selected independently. This distinguishes the present method from glass composites prepared by melt-quenching followed by annealing-induced crystallization of scintillating particles within the glass volume. While the latter approach requires the glass composition to be compatible with subsequent crystallization of the desired scintillating phase, the present method allows pre-prepared scintillators and glass matrices with substantially different elemental compositions to be combined in a single processing step. This provides considerably greater freedom in tailoring the composite material to the requirements of a particular application.

## Author contributions

Herbert Kindl: investigation, methodology, project administration, visualization, writing – original draft, writing – review & editing; František Zajíc: investigation, methodology, project administration, visualization, writing – original draft, writing – review & editing; Eduard Leitner: investigation, methodology; Karol Bartosiewicz: methodology, supervision, writing – review & editing; Vítězslav Jarý: supervision, validation; Monika Kotyková: investigation; Romana Kučerková: investigation; Alena Beitlerová: investigation; Paweł Socha: resources; Anton Markovskyi: resources; Ryszard Buczyński: resources; Martin Nikl: conceptualization, funding acquisition, project administration, supervision, writing – review & editing.

## Conflicts of interest

The authors declare that they have no known competing financial interests or personal relationships that could have appeared to influence the work reported in this paper.

## Data Availability

The data supporting this article are available at the ASEP repository of the Czech Academy of Sciences. https://doi.org/10.57680/asep.0652632.^22^
